# Saccadic “inhibition” unveils the late influence of image content on oculomotor programming

**DOI:** 10.1007/s00221-024-06890-z

**Published:** 2024-07-30

**Authors:** Rebecca Taylor, Antimo Buonocore, Alessio Fracasso

**Affiliations:** 1 School of Psychology, Cardiff University, Tower Building, 70 Park Place, Cardiff, CF10 3AT UK; 2 School of Psychology and Neuroscience, University of Glasgow, Hillhead Street 62, Glasgow, G12 8QE5 Scotland, UK; 3 Department of Educational, Psychological and Communication Sciences, Suor Orsola Benincasa University, Naples, 80135 Italy

**Keywords:** Saccadic inhibition, Faces, Image content, Visual areas, Brainstem

## Abstract

Image content is prioritized in the visual system. Faces are a paradigmatic example, receiving preferential processing along the visual pathway compared to other visual stimuli. Moreover, face prioritization manifests also in behavior. People tend to look at faces more frequently and for longer periods, and saccadic reaction times can be faster when targeting a face as opposed to a phase-scrambled control. However, it is currently not clear at which stage image content affects oculomotor planning and execution. It can be hypothesized that image content directly influences oculomotor signal generation. Alternatively, the image content could exert its influence on oculomotor planning and execution at a later stage, after the image has been processed. Here we aim to disentangle these two alternative hypotheses by measuring the frequency of saccades toward a visual target when the latter is followed by a visual transient in the central visual field. Behaviorally, this paradigm leads to a reduction in saccade frequency that happens about 90 ms after any visual transient event, also known as saccadic “inhibition”. In two experiments, we measured occurrence of saccades in visually guided saccades as well as microsaccades during fixation, using face and noise-matched visual stimuli. We observed that while the reduction in saccade occurrence was similar for both stimulus types, face stimuli lead to a prolonged reduction in eye movements. Moreover, saccade kinematics were altered by both stimulus types, showing an amplitude reduction without change in peak velocity for the earliest saccades. Taken together, our experiments imply that face stimuli primarily affect the later stages of the behavioral phenomenon of saccadic “inhibition”. We propose that while some stimulus features are processed at an early stage and can quickly influence eye movements, a delayed signal conveying image content information is necessary to further inhibit/delay activity in the oculomotor system to trigger eye movements.

## Introduction

Image content is prioritized at the behavioral and neural level (Thorpe and Fabre-Thorpe 2001; Rousselet et al. 2002). Faces are a notable example, and previous studies indicate the existence of dedicated face processing mechanisms in human and non-human primates (Kanwisher et al. 1997; McCarthy et al. 1997; Haxby et al. 2000; Tsao and Livingstone 2008; Rossion et al. 2012), possibly due to the evolutionary relevance of recognizing conspecifics and in-group members (Goren et al. 1975; Johnson 2005).

The prioritization of face processing is not limited to upstream, high-order visual processing areas (Kanwisher et al. 1997) but can be observed also in the motor domain. One example is the superior colliculus (SC), an area in the midbrain critically involved in eye movement planning and execution (Hafed et al. 2023). Neurons in the superficial layers of SC show a preferential response to face-like images, indicated by shorter response latencies compared to scrambled stimuli (Nguyen et al. 2014). Moreover, recent evidence shows that neurons in the superficial and intermediate layers of SC can exhibit stronger responses to objects than to visual noise stimuli (Bogadhi and Hafed 2023), suggesting that the SC may possess a generalized object detection capability, potentially linked to the low spatial frequency sensitivity in SC (Chen and Hafed 2017, 2018; Chen et al. 2018; Bogadhi and Hafed 2023; Hafed et al. 2023). Similarly, neurons in the amygdala exhibit visual responses to face stimuli (McFadyen et al. 2017), and processes visual information with low-frequency content (Chen et al. 2018).

The existence of a subcortical pathway linking the retina to the superior colliculus with projection to the pulvinar nucleus and the amygdala is corroborated by structural and functional data (Burton and Jones 1976; Benevento and Standage 1983; Tamietto et al. 2012; Rafal et al. 2015). This route appears to be involved in processing the coarse visual information that constitutes faces (Morris et al. 2001; Vuilleumier et al. 2003) that can be used as a quick “face detection” mechanisms (Johnson 2005).

Face prioritization manifests also in oculomotor responses. People tend to look at faces more frequently and for longer periods (Buswell 1935; Yarbus and Yarbus 1967; (Buswell 1935; Henderson 2003; Wade 2020), even when irrelevant (Langton et al. 2008; Sato and Kawahara 2015). In oculomotor behaviour, saccadic reaction times are shorter when targeting a face as opposed to a phase-scrambled control (Kirchner and Thorpe 2006; Crouzet and Thorpe 2011; Buonocore et al. 2020; Webb et al. 2022), and microsaccades can be biased towards faces within 100 ms after image onset (Bogadhi et al. 2020).

While convincing evidence exists showing the neural and behavioural prioritization of image content in general, and faces in particular (Kanwisher et al. 1997; Thorpe and Fabre-Thorpe 2001; Rousselet et al. 2002; Tsao and Livingstone 2008), it is currently not clear at which stage image content affects oculomotor planning and execution. It can be hypothesized that (i) image content influences oculomotor signal generation at an early stage. Alternatively, (ii) the influence of image content could take place only at a later stage, after the image has been processed.

Here we aim to disentangle these two alternative hypotheses by leveraging an oculomotor phenomenon called saccadic “inhibition” (Reingold and Stampe 1999, 2002; Buonocore and McIntosh 2008). Behaviorally, it has been observed that the onset of a visual transient during oculomotor programming can disrupt the generation of eye movements, leading to delay and sometimes cancellation of an impending saccade. Saccadic “inhibition” is characterized by a marked decrease in saccade frequency, starting approximately 90 ms after visual transient onset (Buonocore and McIntosh 2008; Edelman and Xu 2009; Bompas and Sumner 2011). Please note that we are using quotation marks (“inhibition”) when referring to the behavioral phenomenon, showcased by a decrease in saccade frequency.

Previous research has demonstrated that both the latency and magnitude of saccadic “inhibition” are sensitive to low-level stimulus characteristics such as stimulus contrast, size (Bompas and Sumner 2011; Buonocore and McIntosh 2012, 2013; Bonneh et al. 2015; Khademi et al. 2023), spatial frequency (Bonneh et al. 2015), orientation, motion direction, and motion speed (Khademi et al. 2023), as well as high-level processes such as attentional allocation (Reingold and Stampe 2004; Buonocore and McIntosh 2012, 2013) and stimulus familiarity (Kadosh and Bonneh 2022).

The onset of saccadic “inhibition” can be as short as 50 ms in monkeys (Khademi et al. 2023) and less than 60 ms in humans (Bompas et al. 2024). This delay is comparable to the neural delay in transmitting signals from the retina to the SC via the retinotectal pathway, which has been estimated in 34–45 ms in monkeys (Rizzolatti et al. 1980; Chen et al. 2018; Hafed et al. 2023). Despite the name, the term “inhibition” only refers to the observed oculomotor phenomenon, not its neural mechanisms, which are still debated. At present, the most convincing evidence suggests that the mechanisms underlying saccadic “inhibition” might be closely linked to the final oculomotor centres involving the SC and the brainstem. One possible explanation of the effect does not involve an inhibition of saccade generation insofar as the presentation of the visual transient would desynchronize the action potentials emitted by saccade-related neurons in the deep superior colliculus, delaying the triggering of the saccade (Goffart et al. 2017). Alternatively, another possible mechanism involves the recruitment of omnipause neurons (OPNs) located in the nucleus raphe interpositus (Büttner-Ennever et al. 1988; Langer and Kaneko 1990; Horn et al. 1994). OPNs are in fact a class of tonic neurons that lie in the midbrain very close to the midline which fire steadily during fixation and stop their activity during saccades (Cohen and Henn 1972; Luschei and Fuchs 1972; Keller 1974; Evinger et al. 1982), suggesting an involvement in the triggering of eye movements. Sudden reactivation of OPNs by direct visual stimulation (Buonocore and Hafed 2021) might consequently delay the movement (Buonocore and Hafed 2023).

It is important to know that this mechanism might involve also other populations of neurons within the brainstem, since neither fixation instability nor change in saccade onset were observed following a lesion or inactivation of the nucleus raphe interpositus in non-human primates (Kaneko 1996; Soetedjo et al. 2002). The inhibitory influence of OPNs on saccade generation is still hypothetical, as there is no direct evidence that inactivation of OPNs affects fixation (Krauzlis et al. 2017). The observed slowing of saccades during microstimulation of the raphe interpositus may result from inhibitory synapses between OPNs and premotor burst neurons. This pause likely synchronizes horizontal and vertical saccade onsets by projecting to burst neurons in the PPRF and RIMLF (Goffart et al. 2024; Ohgaki et al. 1989).

Nonetheless, neuropsychological observations relate the opsoclonus syndrome, i.e. the erratic eye movement behavior without intersaccadic intervals (e.g. Kilgo and Schwartze 1984), to a dysfunction within the brainstem at the level of the nucleus raphe interpositus, suggesting the idea that this region contains elements involved in maintaining stage gaze direction (Takahashi et al. 2022). If an inhibition takes place during the final stage of the oculomotor circuitry, it might efficiently interrupt an impending saccade, allowing the time for voluntary control to take place and reprogram the saccade (Buonocore et al. 2017b; Bompas et al. 2020). Moreover, OPNs might still be activated via anatomical projections from the rostral SC (Büttner-Ennever et al. 1988), inhibiting the premotor burst neurons in the pontine and mesencephalic reticular formations.

**Fig. 1:**
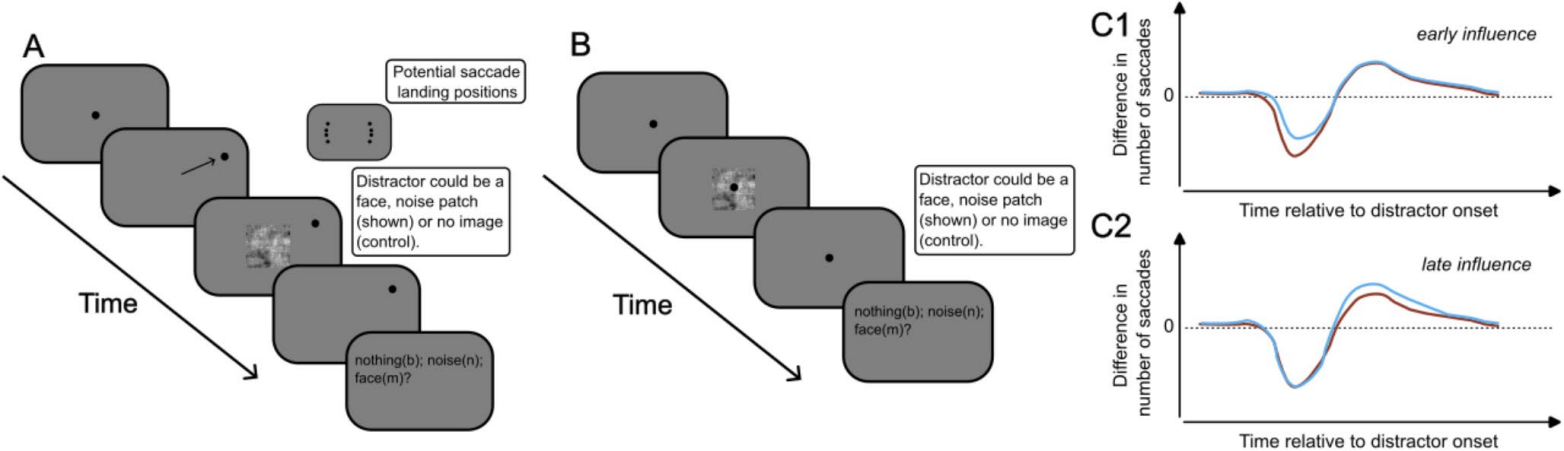
Experimental procedure and hypotheses. **(A)** Procedure for experiment 1 (visually-guided saccades). The eight potential saccade landing positions can be seen in the upper right-hand portion of the panel. **(B)** Procedure for experiment 2 (microsaccades). In both Fig. 1A and B, the example trial shown is a noise-matched condition. **C1/C2.** Experimental hypotheses on the influence of image content on saccadic “inhibition” profiles. Blue and red curves indicate saccadic “inhibition” profiles obtained from images with different content. **C1**, early influence of image content on saccadic “inhibition”. **C2**, late influence of image content on saccadic “inhibition”

In the experiments that follow, we recorded saccade latencies in response to face and noise-matched stimuli to estimate the so-called saccade inhibition from the latency, duration and amplitude of saccades. We hypothesized that if image content is processed at the level of structures where saccadic “inhibition” originates, such as the superior colliculus or the brainstem, it should modulate the onset of saccadic “inhibition” (Khademi et al. 2023), possibly anticipating it (Fig. 1, panel C_1_). Conversely, if later stages of inhibition are affected, it would suggest that the influence of stimulus content has been relayed only once the image has been processed by higher-order visual areas (Kanwisher et al. 1997; Tsao et al. 2008), (Fig. 1, panel C_2_).

This latter view would be supported by previous findings relating higher level cortical processing, such as allocation of visual attention in the direction of an upcoming saccade, to modulations of saccadic “inhibition” duration and magnitude but not its onset (Reingold and Stampe 2004; Buonocore and McIntosh 2013). Here, we address these questions in two experiments designed to test the role of face stimuli in modulating different stages of saccadic “inhibition” profile. The modulation of saccadic “inhibition” is examined in a voluntary task by means of visually guided saccades as well as in a fixation task, examining microsaccades.

## Materials & methods

### Participants

28 participants aged 18–48 took part in Experiment 1 (saccades measurement). Of these 28 participants, 23 participated in Experiment 2 (microsaccades measurement). Participants were recruited through the University of Glasgow participants pool and had normal or corrected to normal vision. Written informed consent was obtained, in accordance with the 1964 Declaration of Helsinki. Subjects received a compensation of £6 per testing hour. Ethical approval was granted by the local ethics committee at the college of Medical, Veterinary and Life Sciences, University of Glasgow.

### Apparatus

Participants were placed in a chin- and forehead rest to ensure head stability. Responses were given by pressing keys on a standard keyboard. Stimuli were presented on a 24-inch LCD monitor (1920 × 1024 pixels) at 144 Hz. Display luminance was linearized. Participant’s eyes were aligned with the centre of the screen at a distance of 64.5 cm. Eye position was sampled using an Eyelink 1000 (SR Research, Ltd., Ottawa, ON), acquiring data at 1000 Hz. A five-point calibration sequence in a square-shaped pattern was performed at the beginning of each experimental block. Both eyes were tracked for the duration of the experiment. The experiment was programmed in Matlab (R2021a, The Math Works, Inc., 85 Natick, MA), with the Psychtoolbox (Brainard, 1997) and the Eyelink toolbox (Cornelissen, Peters, & 86 Palmer, 2002).

### Visual stimuli and experimental conditions

The procedure consisted of two experiments. Each experiment consisted of three conditions: a *control condition*, in which no image was presented, a *face condition*, in which an image representing a human face was presented, and a *noise condition* where a scrambled version of the face stimuli was presented (see below for the process we adopted to equate the low-level visual features between *face and noise conditions*). Importantly, the images in the *noise condition* retained the low-level visual information of the face images (total root mean square – RMS – contrast and spatial frequency content) but were not recognizable as faces. The procedure to equate the low-level visual features in the *face and noise conditions* was the following: we used a total of 10 grayscale images representing human faces: images were obtained from previously published studies (Tsao et al., 2006; Boghadi et al., 2019; Boghadi et al., 2020; Boghadi & Hafed, 2022). The luminance histograms and spatial frequency spectra of the 10 face images were iteratively equalized using the SHINE toolbox (Willenbockel et al., 2010). Specifically, we ran 20 iterations of histogram matching (*histMatch* function) of the gray levels across the face images, as well as spectral matching across the same images (*specMatch* function). To generate phase-scrambled images, we randomized the phase matrices of the Fourier-decomposed images, while keeping the amplitude matrices unchanged. Then, to match the real and phase-scrambled images further, we took all face images and their corresponding phase scrambled images, and we again iteratively matched them once more for histogram levels and frequency spectra using the same SHINE toolbox functions (again, with 20 iterations).

### Procedure

#### Saccade reaction time estimation

We measured participants’ median saccadic reaction during the first part of the experiment. Participants completed 45 trials where they fixated on a central stimulus before pressing the spacebar to initiate the trial. The fixation point was a black circle measuring 0.5 degrees of visual angle. After a random temporal interval between 900 and 1500ms from pressing the spacebar, the fixation point shifted 10 degrees of visual angle either left or right along the horizontal axis. Participants were asked to follow the fixation point with their eyes. A gaze-contingent algorithm estimated participants’ reaction time on each trial (Fracasso et al. 2015; Fabius et al. 2016; Fracasso and Melcher 2016; Buonocore et al. 2017a, b, c). The median saccadic reaction time among the 45 trials was computed for each participant taking part in the measurement and stored for later use.

#### Experiment 1

In Experiment 1 (saccades), participants were asked to fixate a central stimulus identical to the one used during the estimation of median saccade reaction time. Each trial started with the presentation of a fixation point. Participants were required to fixate and press the spacebar to initiate each trial. Between 900 and 1500ms later, the fixation point shifted 10 degrees of visual angle across the screen to one of eight potential locations. The potential peripheral targets were placed 5 and 15 degrees above and below the horizontal meridian on either side of the screen (see Fig. 1A). In the *control condition*, a uniform texture with the same color as the screen background was presented at a given inter-stimulus-interval (ISI) from peripheral target onset. The ISI was selected randomly from a uniform distribution ranging between 10% and 60% of individual median saccade reaction time (Fracasso et al. 2010, 2013; Melcher and Fracasso 2012). The control condition was necessary to obtain an empirical distribution of saccadic reaction times that could be compared with the *distractor conditions*. In the two *distractor conditions* (*face and noise*), a transient image was presented in the center of the screen at a variable interval after the onset of the peripheral target (size = 2.5 × 2.5 degrees of visual angle, duration = 41 msec, 6 frames at 144hz), using the same criteria adopted for the *control condition*. Participants were instructed to perform a saccade towards the peripheral target, attempting to ignore any potential distractors. After each trial, they were asked to indicate via key press whether a face, noise, or no image had been presented. Each block in Experiment 1 comprised 50 trials.

#### Experiment 2

In Experiment 2 (microsaccades), the procedure was identical to Experiment 1. The only difference was that the starting fixation point did not shift to the left/right of the screen, but remained in the center of the screen throughout the trial (Fig. 1B). Each block in Experiment 2 comprised 50 trials. Each participant completed two one-hour measurement sessions, on non-consecutive days. Across the two sessions, participants completed a variable number of blocks (ranging between 10 and 18), alternating between the microsaccade and saccade measurements. Each block consisted of 50 trials and lasted approximately 5 min. Between each block, participants were asked if they would like a break to rest. At the beginning of each block, a five-point calibration on the horizontal and vertical axes was performed.

### Data analysis

Eye traces (x and y positions over time) for each trial were analyzed and parsed in Matlab (mathworks.com, version 2019b), using the toolbox provided by Nyström and Holmqvist (2010). Data from the left and right eyes were analyzed independently. We considered a saccade as successfully detected if an eye movement was detected in both eyes (valid saccade). In the rest of the manuscript, we will refer to ‘valid saccades’ as ‘saccades’. Saccades detected only in one eye were considered noise and not analyzed further. For each saccade, we extracted peak velocity, amplitude and duration and stored these values for later analysis. Data analysis was carried out in R. For each experimental condition (*control*,* face*,* noise*) we computed the delay between saccade onset and distractor onset (saccade onset with respect to distractor onset). We used paired t-tests and t-tests to perform statistical analysis comparing the different experimental conditions. We used the false discovery rate (FDR) method to correct for multiple comparisons at a p < 0.05 level.

#### Experiment 1 (saccades)

Trials were filtered according to the following criteria: saccadic amplitude between 4 and 16 degrees of visual angle, saccade onset with respect to distractor onset between − 20 and 320ms, saccade reaction time between 80-550ms, peak velocity thresholded adaptively between 0.05th-95th percentile, and saccade duration longer than the first percentile of the participant saccade duration distribution, and shorter than 100ms (Fabius et al. 2019, 2022; Fracasso et al. 2023). This resulted in the exclusion of 13% of trials on average across participants. Saccade onset times with respect to distractor onset were binned per participant and condition into 20ms time bins (from − 20ms to 320ms). Next, the number of saccades performed per participant per condition in each time bin was counted. To compute the proportion of saccades per bin, we divided the number of saccades performed per participant per condition in each time bin by the total number of saccades performed per participant across all time bins, per condition. Proportions were averaged across participants to compute the mean number of saccades performed per condition in each time bin. Paired t-tests were performed across time bins between noise and face conditions. We used the false discovery rate (FDR) method to correct for multiple comparisons at a *p* < 0.05 level.

The difference between the face and control experimental conditions as well as between noise and control experimental conditions were calculated by subtracting the proportion of saccades performed in each time bin in the control condition from the proportion of saccades performed in each experimental condition in the corresponding time bin. This was first done at an individual participant level then averaged across all participants to give the mean difference per experimental condition in each time bin. Paired t-tests with FDR corrections were performed between face and noise conditions per time bin.

Next, we calculated the average saccadic amplitude, peak velocity and duration per participant per time bin in each condition. Given the relatively low number of saccades performed in some temporal bins, we used 30ms time bins rather than 20ms bins to increase the number of saccades per bin. We then calculated the mean saccadic amplitude, peak velocity and duration per temporal bin and participant, in each experimental condition. t-tests with FDR corrections were performed between each of the three conditions across each time bin.

We computed the radial eye position taking the Euclidean distance of any eye position sample during a given movement from the eye position at saccade onset, that is, the angular distance travelled by the eyes from saccade onset. In this way we could plot a single trace to demonstrate saccade amplitude over time, instead of having two separate traces (one for horizontal eye position and one for vertical eye position). We computed radial velocity by taking the first derivative of radial eye position.﻿

#### Experiment 2 (microsaccades)

Microsaccades were identified as eye movements which were performed up to 200ms before and 800ms after the presentation of the distractor, and which met the following criteria: saccadic amplitude between 0.05 and 1.5 degrees of visual angle, and duration between 0.005 and 0.08s.

Saccades were binned into 50ms time bins with respect to the onset of the distractor. From here, the average number of saccades, proportion of saccades and differences between conditions and control were calculated in the same manner as in the saccade experiment. For the kinematics analysis, we used 60ms bins, and calculated average peak velocity, saccade amplitude and saccade duration according to the same method as in the saccade experiment. Paired t-tests with FDR correction were used to analyse the differences in the number of saccades in each condition, and t-tests with FDR correction were used to analyse the kinematic data.

#### Experiment 1&2 comparison

Lastly, we were interested in comparing the effects of stimulus type on visually-guided saccades and microsaccades. We first added the differences between the control and face conditions in the microsaccade experiment in each time bin along the span of microsaccade “inhibition”: 50ms and 350ms. Next, we added the differences between the control and face conditions in the saccade experiment in each time bin along the span of saccade “inhibition”: between 50ms and 150ms. We repeated this procedure to estimate the difference in the “inhibition” strength between saccades and microsaccades in the noise condition.

To compare the magnitude of the categorical effect between visually-guided saccades and microsaccades, we subtracted the difference between the noise and control conditions from the difference between face and control conditions in the microsaccade experiment between in each time bin 50ms and 350ms, then summed the results. We repeated this procedure in the saccade experiment, this time between 50ms and 150ms.

We tested the relationship of saccadic “inhibition” magnitude between visually guided saccades and microsaccades using a linear model. We assessed potential outliers by obtaining leverage estimates from the hat-value for each observation from the full model (the model containing all statistical units - participants). We estimated the weight of each hat-value by deriving the corresponding studentized residual. Overall, we observed 1 hat-value whose studentized residual fell beyond the 95% confidence interval; Table 1.

## Results

### Experiment 1 (saccades)

**Fig. 2:**
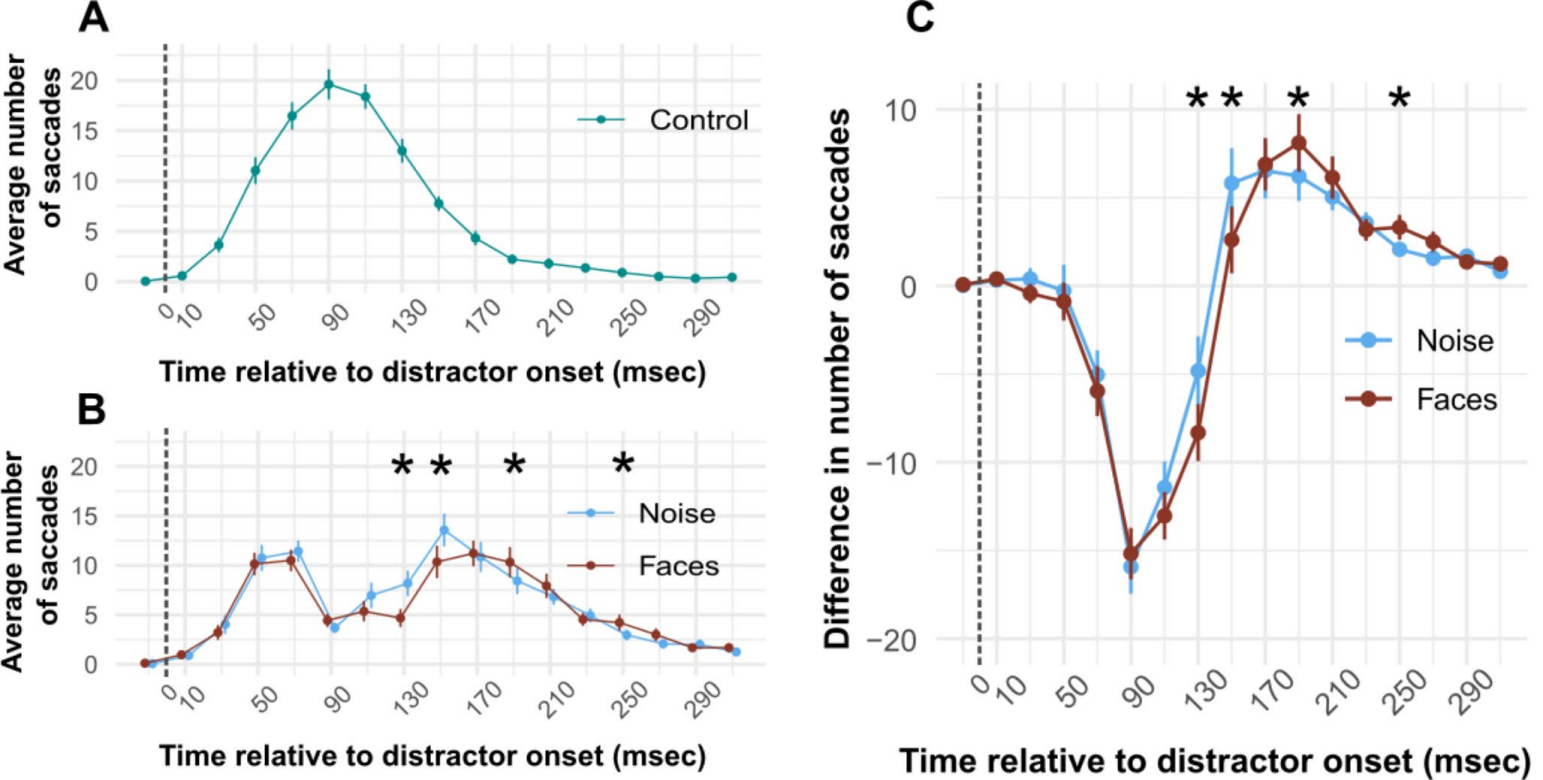
Average number of saccades across time. **(A)** Control condition. time is measured from the onset of the distractor, and saccades are binned into 20ms time bins. Each time bin is represented by its midpoint (i.e. 10ms represents all saccades occurring in the 0-20ms time bin). **(B)** Same as A, the noise and face conditions are reported. **(C)** Difference in the number of saccades between the noise and control conditions (blue) and the difference in the number of saccades between the face and control conditions (red). Asterisks indicate time bins where results of paired t-tests between the face and noise conditions were statistically significant after FDR correction. Error bars represent +/- standard error of the mean. Dashed lines indicate the distractor onset. Our results indicate a late influence of image content on saccade “inhibition” profile

We observed the expected saccade occurrence following the presentation of a high contrast visual stimulus. Compared to the control condition (Fig. 2A), the frequency of visually guided saccades started decreasing sharply around 75ms after stimulus onset (Fig. 2B).

The average number of performed saccades dropped from ~ 20 (range [14–25]) to ~ 5 (range [1 8]).

Crucially, we observed significant differences in saccadic “inhibition” profiles between faces and scrambled, matched-control stimuli, with the former leading to significantly less numerous saccades than the latter. Figure 2A and B show the average number of saccades over time, measured from the onset of the distractor. Paired t-tests revealed statistically significant differences between the number of saccades observed in the noise and face conditions in the following time bins: 120ms-140ms: *t*(27) = 4.153, *p* = 0.004; 140ms-160ms: *t*(27) = 3.982, *p* = 0.004; 180ms-200ms: *t*(27) = -2.972, *p* = 0.035; 240ms-260ms: *t*(27) = -2.795, *p* = 0.040. (p-values are FDR corrected).

Figure 2C shows the average difference in the number of saccades in the noise and face conditions compared to the control condition over time. As this represents the same data reported in Fig. 2B, minus a constant (control condition, Fig. 2A), the statistical results are identical as those reported above for Fig. 2B.

The same results were observed when expressing saccadic “inhibition” profiles in terms of saccade proportion instead of the absolute number of saccades (not shown).

Kinematics of visually guided saccades (specifically, saccade amplitude) is also significantly affected by the presence of a visual distractor.

**Fig. 3:**
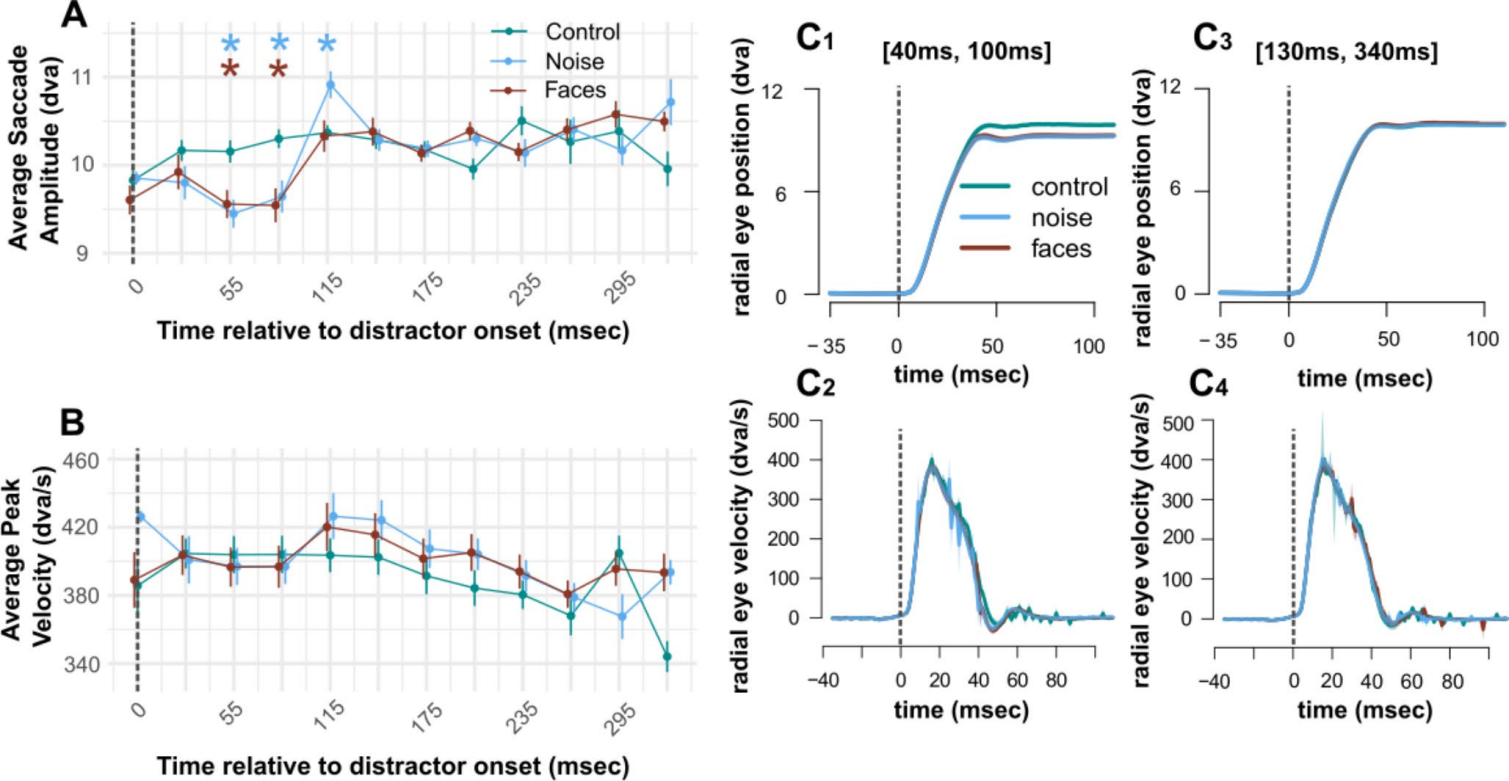
Saccade Kinematics. **(A)**. shows the average saccadic amplitude over time across experimental conditions. Time is measured from distractor onset. Saccades are binned into 30ms time bins. All time bins are represented by their midpoint (e.g. 55 represents all saccades performed in the 40-70ms time bin). Error bars represent +/- standard error of the mean. **(B)**. shows the average peak velocity over time. t-tests revealed no significant differences in saccadic amplitude or peak velocity between the face and control conditions. The blue asterisks show statistically significant differences between the noise and control conditions, and the red asterisks represent statistically significant differences between the face and control conditions. All p-values are FDR corrected. Figure 2C: Radial eye position and radial eye velocity over time. **(C)**. Average radial eye position and velocity. Figure 2C1 and 2C2 shows radial eye position and radial eye velocity respectively over time for saccades performed between 40 ms and 100 ms after distractor onset. Figure 2C3 and 2C4 shows radial eye position and radial eye velocity respectively over time for saccades performed between 130 ms and 340 ms after distractor onset. Dashed lines indicate the distractor onset

Figure 3A shows the average saccadic amplitude in each experimental condition over time. Results of t-tests were statistically significant when comparing average saccade amplitude in the noise condition compared to the control condition in the following time bins: 40-70ms: *t*(27) = 3.495, *p* = 0.012; 70-100ms: *t*(27) = 3.130, *p* = 0.017; 100-130ms: *t*(27) = -3.050, *p* = 0.017. Results of t-tests were statistically significant when comparing average saccade amplitude in the face condition compared to the control condition in the following time bins: 40-70ms: t(27) = 2.943, *p* = 0.029 70-100ms: *t*(27) = 3.448, *p* = 0.016. Overall, when a significant difference was detected with respect to control condition, saccade amplitude was reduced by about 5%, from an average of 10.25 dva to 9.65 dva (range [9.20 9.92] dva).

All p-values are FDR corrected. Figure 3, panelC_1_ shows the differences between radial eye position in saccades performed between 40-100ms in the noise and face conditions compared to the control condition. Figure 3, panelC_3_ shows how in time bins which did not yield significant differences in saccadic amplitude, radial eye position does not differ between the conditions.

### Experiment 2 (microsaccades)

**Fig. 4:**
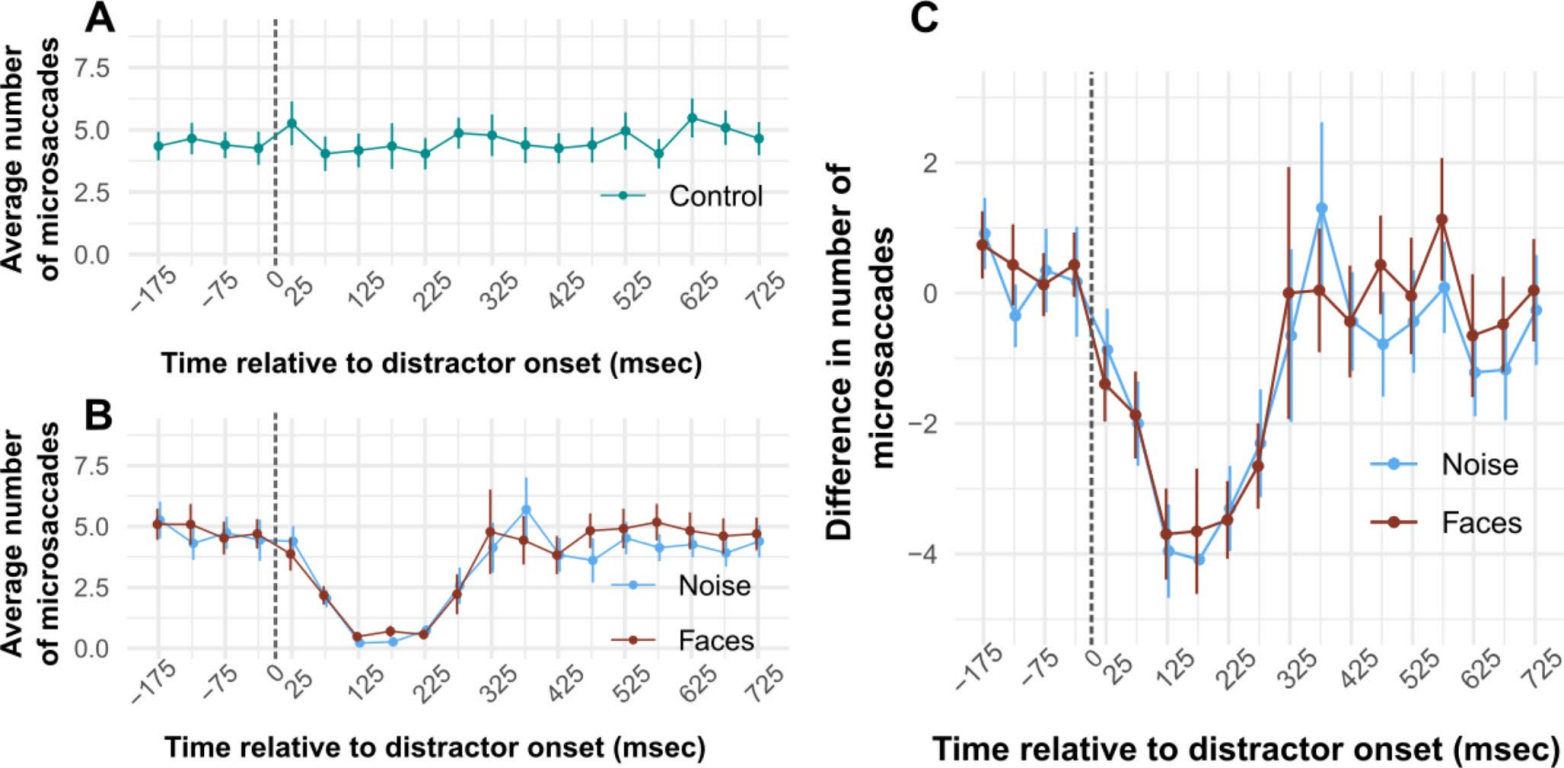
Average number of microsaccades across time. **(A)**. Noise matched-control stimuli. Time is measured from the onset of the distractor, and saccades are binned into 50ms time bins. Each time bin is represented by its midpoint (i.e. 125ms represents all saccades occurring in the 100-150ms time bin).**(B)**. Same as A, for face and noise conditions.**(C)**. Difference in the number of microsaccades between the noise and control conditions (blue) and the difference in the number of saccades between the face and control conditions (red). Error bars represent +/- standard error of the mean. Dashed lines indicate the distractor onset

In experiment 2 we investigated the generation of microsaccades following the presentation of a high contrast visual stimuli (Engbert and Kliegl 2003; Rolfs et al. 2008; Hafed and Ignashchenkova 2013; Buonocore et al. 2021). Compared to the control condition (Fig. 4A), the frequency of microsaccades started decreasing sharply around 50ms after stimuli onset (Fig. 4B). In this case however, we did not observe significant differences between the faces and scrambled, matched-control stimuli.

Figure 4A and B show the average number of microsaccades over time, measured from the onset of the distractor. Figure 4C shows the average difference in the number of microsaccades in the faces and scrambled, matched-control stimuli over time. Paired t-tests were performed between the noise and face conditions across all time bins for both the number of saccades and the differences in the number of saccades. After FDR correction, none of the comparisons yield statistically significant difference in either the number of saccades or difference in number of saccades. The average number of performed microsaccades dropped from ~ 5 (range [3–7]) to less than 1 (range [0 1]).

Virtually identical results were observed when expressing saccadic “inhibition” profiles in terms of microsaccade proportion instead of the absolute number of microsaccades (not shown).

**Fig. 5:**
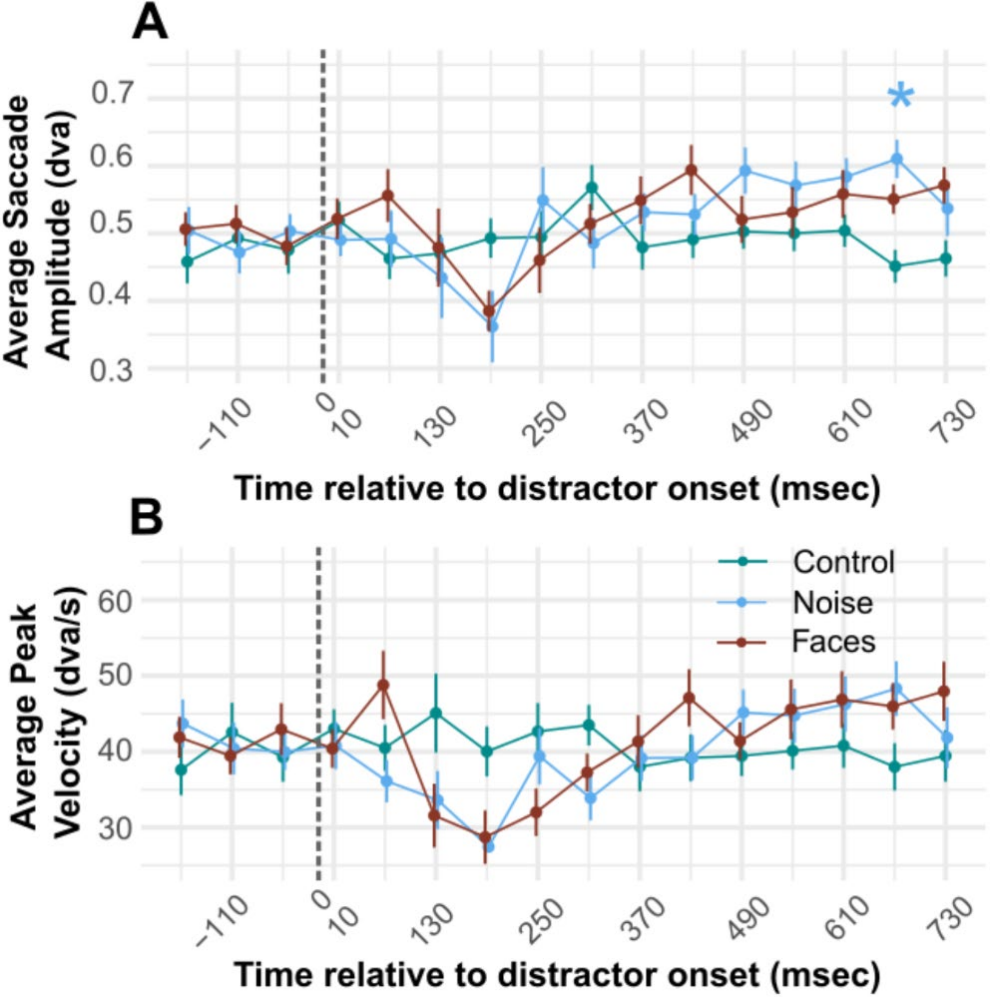
Microsaccade kinematics. **(A)** average saccadic amplitude over time. Saccades are binned into 60ms time bins. All time bins are represented by their midpoint (e.g. 130 represents all saccades performed in the 100-160ms time bin). Error bars represent +/- standard error of the mean. t-tests between all conditions only revealed one statistically significant difference: between saccadic amplitude the control and noise conditions in the 640-700ms time bin. This is represented in Fig. 6A by a blue asterisk. **(B)** Shows the average peak velocity over time. Dashed lines indicate the distractor onset

Microsaccade kinematics are not significantly affected by the presence of a visual distractor. Although a visible decrease in microsaccade amplitude and velocity can be appreciated in Fig. 5A&B, the comparison with control condition did not yield significant differences after correction, except for one point later in the profile.

Figure 5A shows the average saccadic amplitude in each experimental condition over time. Results of t-tests were statistically significant when comparing average saccade amplitude in the noise condition compared to the control condition in the 640-670ms time bin only: *t*(22) = -4.122, *p* = 0.003. Figure 5B shows the average peak velocity in each experimental condition over time. After FDR correction, t-tests did not yield significant differences between experimental conditions over time.

### Experiment 1&2 comparison

**Fig. 6:**
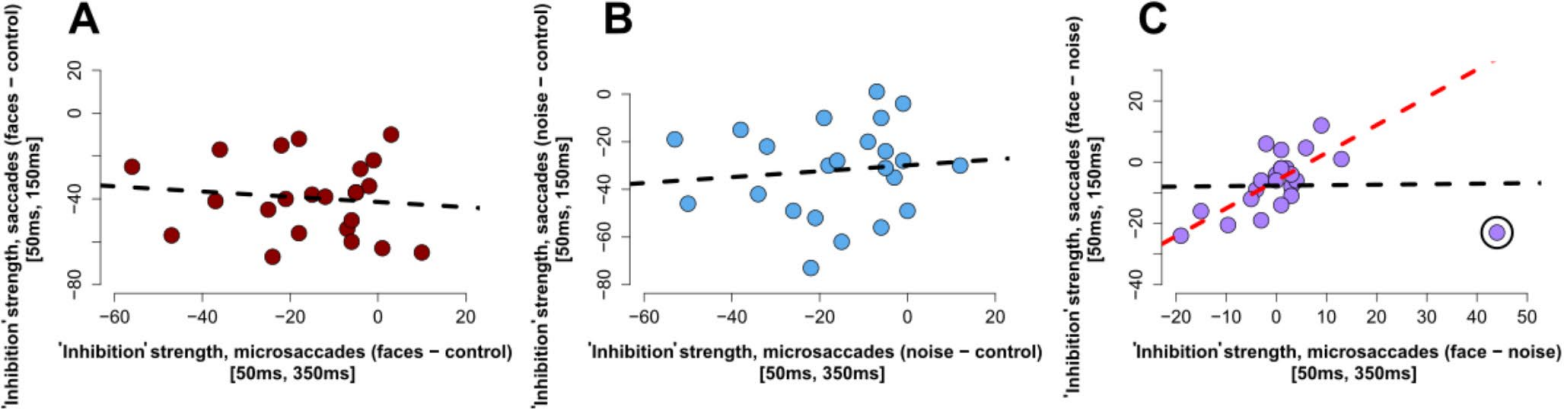
Strength of saccadic “inhibition” **(A)** Scatterplot of “inhibition” effect magnitude in the face condition between microsaccades (Experiment 2, x-axis) and saccades (Experiment 1, y-axis). Data from the 23 participants that took part in both experiments is reported. The black dashed line shows the fitted regression line, not significant. **(B)** Same as panel A, noise condition. The black dashed line shows the fitted regression line, not significant. **(C)** Scatterplot of categorical effect magnitude for microsaccades (Experiment 2, x-axis) and saccade (Experiment 1, y-axis). The black dashed line shows the fitted regression line, not significant. When excluding the individual with the stronger leverage influence (circled in black) we observe a significant linear trend (red dashed line). We assessed leverage by deriving the hat-value for each observation from the full model (the model containing all statistical units - participants). We estimated the weight of each hat-value by deriving the corresponding studentized residual. Overall, we observed 1 hat-value whose studentized residual fell beyond the 95% confidence interval

The changes in the number of saccades toward the face and scrambled stimuli (Experiment 1) were not associated with changes in the number of microsaccades (Experiment 2) (Fig. 6, A&B). However, we observed a relationship in the effect of image content between visually guided saccades (Experiment 1) and microsaccades (Experiment 2) during saccadic “inhibition” window (Fig. 6C; Table 1).

**Table 1:** The results for the linear models shown in Fig. 6 are summarized in the table below:

Experimental condition	*R* ^2^	b coefficient	degrees of freedom	t-statistic	*p*-value
Faces	0.010	-0.121	22	-0.529	*n.s.*
Noise	0.010	0.123	22	0.492	*n.s.*
Categorical Effect – *before* outlier exclusion	0.003	0.016	22	0.091	*n.s.*
Categorical Effect – *after* outlier exclusion	0.419	0.907	21	3.804	0.001

## Discussion

Here we demonstrate a distinct an effect of the distractor image content on the generation of saccades (Fig. 4B and C): presenting a human face at fixation during oculomotor programming toward a lateralized target led to stronger saccadic “inhibition” compared to a scrambled image with identical spatial frequency and contrast. This observation is in line with prior research showing that stimulus characteristics can affect oculomotor programming and that they can be reliably detected by inspection of the latency distribution of saccades (Khademi et al. 2023). For example, the intrinsic features of the stimulus, such as its size, spatial frequency, contrast, orientation, and motion direction have been found to significantly modulate both the magnitude of “inhibition” and its latency (Reingold and Stampe 1999, 2000; Stampe and Reingold 2002; Reingold and Stampe 2004; Rolfs et al. 2008; Bompas and Sumner 2011; Buonocore and McIntosh 2012; Buonocore and McIntosh 2013; Bonneh et al. 2015; Khademi et al. 2023). Our study expands on these findings by illustrating that image content can also modulate the saccadic “inhibition” profile, similarly to other cognitive processes, for example attention allocation (Reingold and Stampe 2004; Buonocore and McIntosh 2013) and familiarity with the stimulus (Kadosh and Bonneh 2022).

In Experiment 1, the difference between face and scrambled, matched-control stimuli, become apparent at a later stage of oculo-motor programming. That is, the difference between Face and Noise condition is not apparent as soon as the occurrence of saccades starts to be reduced compared to the Control condition, but becomes statistically significant later (Fig. 4B, C). In other words, the latency of the influence of the flashed distractor per se was not affected by stimulus type, contrary to what it has been observed for other stimulus characteristics such as size, spatial frequency, contrast, orientation, motion direction, and motion speed (e.g. Khademi et al. 2023). We can speculate that information about image content increased stimulus saliency but only after visual processing in higher order visual areas.

In Experiment 2 we observed a reduction in the rate of microsaccades induced by the same stimuli, however we did not observe significant effect of image content. There are different, non mutually-exclusive accounts for the discrepancy between Experiment 1 and 2. First, we observe strong reduction in the number of microsaccades. Microsaccades were virtually absent during an interval ranging from 100 to 250ms after the onset of the distractor (Fig. 3B). This leads to a decrease in the dynamic range available to observe any modulation of the influence of the transient stimulus depending upon its image content, essentially leading to a floor effect. Second, in Experiment 1 saccades were visually-guided, possibly leading to greater participant engagement and increased sensitivity to image content. Nonetheless, we provide indirect evidence suggesting that the oculo-motor system is sensitive to image content also for microsaccades. We observed a correlation between the image content effect during saccadic “inhibition” in Experiment 1 and Experiment 2 (Fig. 5C), indicating a relationship in the effect of image content between visually guided saccades (Experiment 1) and microsaccades (Experiment 2).

In our analysis of saccade kinematics, we corroborated earlier findings showing how irrelevant visual stimuli can affect ongoing saccades (Guillaume 2012; Buonocore et al. 2016, 2017a). Notably, in Experiment 1, we observed a significant decrease in saccadic amplitude as soon as 50 ms following the onset of a distractor, with this effect lasting up to 100 ms after the distractor onset. This reduction in saccadic amplitude occurred for both experimental conditions (Face or Noise) and amounted to about 5% of intended saccade amplitude. Our data suggests a divergence from the typical eye movement main sequence (Bahill et al. 1975), as the average peak velocity during this interval remained unchanged by the distractor, compatible with the existing literature (Buonocore et al. 2016, 2017a). This implies that while saccade’s velocity was consistent with the planned eye movement for the peripheral target eccentricity, the actual amplitude of the saccade was reduced. We acknowledge that small variations in amplitude might also come from curvatures in the trajectories in presence of distractors, as noted in other studies (e.g. McPeek et al. 2003; McSorley et al. 2004). We also noted a similar pattern in microsaccades, although not statistically significant, which contrasts with previous studies showing distinct modulations in microsaccadic movements (Buonocore et al. 2017a). This discrepancy could be due to the limited sensitivity of infrared eye trackers in detecting subtle changes in microsaccade kinematics (Rolfs et al. 2008). Overall, our findings on saccade kinematics indicate that transient visual signals are transmitted to the ocular premotor structures. However, saccade kinematics does not appear to be sensitive to image content. This aligns with the hypothesis that image content modulation operates upstream along the processing hierarchy, impacting later portions of saccades, where kinematics is no longer affected.

The central question remains as to how image content can modulate the activity of oculomotor centers. Studies suggest that the superior colliculus (Nguyen et al. 2014; Bogadhi and Hafed 2023) and other subcortical structures, such as the pulvinar and amygdala (Morris et al. 2001; Vuilleumier et al. 2003; McFadyen et al. 2017), are sensitive to the occurrence of a visual stimulus. Moreover, these areas seem to be responsive also to complex stimuli like objects and faces. If the source of the image content modulation we observed were to originate subcortically, we would expect to see changes soon after the onset of the flashed distractor, as it has been observed in other studies where stimulus features were modulated (Khademi et al. 2023), and possibly leading to stronger changes in saccade kinematics as well (Buonocore et al. 2016, 2017a). However, the changes occurred later, leaving eye movement kinematics unaltered by the image content. Based on the evidence reported, our observations could be better accounted for by visual processing of image content originating from higher-order visual areas, upstream in the visual pathway (Kanwisher et al. 1997; Tsao et al. 2008), then relayed downstream towards the motor output.

In terms of the neural mechanism driving the effect, it has been previously suggested that saccadic “inhibition” might occur at the level of the superior colliculus (Bompas and Sumner 2011; Salinas and Stanford 2018). According to the latest models of saccade generation, populations of neurons in the deep superior colliculus coding for different target eccentricities – including the burst in the rostral portion which codes for microsaccades (Hafed et al. 2009) - are simultaneously activated to maintain an equilibrium of commands that counterbalance with each other during visual fixation (Goffart et al. 2024; Ohgaki et al. 1989). Within the colliculus, active populations of neurons coding for different saccadic goals can inhibit each other by lateral inhibition mechanisms. Moreover, the superior colliculus is sensitive to image content, showing faster responses for visual stimuli with low spatial frequency compared to high spatial frequency, as well as to changes in shape and contrast (e.g. Khademi et al. 2023). This rapid response directly correlates with oculomotor behavior, leading to faster reaction times to more salient stimuli (Olivier et al. 1999). However, the lateral inhibition hypothesis cannot fully account for how saccadic “inhibition” can occur for very small eccentricities and for transients presented directly in the fovea (Engbert and Kliegl 2003; Rolfs et al. 2008; Hafed and Ignashchenkova 2013; Bonneh et al. 2015; Buonocore et al. 2017a). At these small eccentricities the regions activated by the visual stimuli and the oculomotor programming are virtually indistinguishable. Such a large overlap in the visuo-motor representations on the SC surface might prevent inhibitory interactions by adjacent neuronal populations. On the other hand, the delay of saccades can result from a desynchronization of action potentials emitted by saccade-related neurons after the presentation of a new visual transient (Goffart et al. 2017). Weaker synchrony yields scattered and longer saccade reaction times, manifesting as a “dip” in the distribution of saccadic reaction times.

An alternative view posits that the reduction of saccade occurrence involves the reactivation of omnipause neurons (OPNs) in the brainstem as supported by neurophysiological experiments in which electrical stimulation of OPNs delayed the onset of saccades (Gandhi and Sparks 2007). The inhibitory effect could in fact result from following reactivation of the OPNs in response to visual stimulus onsets (Buonocore and Hafed 2023), which seems to be also tuned to stimulus characteristics. While it is unlikely that OPNs directly respond to image content, the underlying inhibitory mechanism might still rely on OPNs reactivation following a visual signal and extending their activity, thereby delaying the execution of eye movements (Gandhi and Keller 1999a, b; Missal and Keller 2002). This perspective is supported by changes in kinematics observed in previous saccadic “inhibition” experiments and in our current study. Specifically, the reactivation of OPNs might truncate the eye movement while maintaining the planned velocity, leading to a deviation from the main sequence (Buonocore et al. 2017a). According to this perspective, the visual activity of OPNs might be further modulated by the strength of the visual signal coming from higher order visual and visuomotor areas and affect saccade triggering even at a later stage of saccade preparation.

## Data Availability

Original data will be made available on reasonable request.
